# Supplementary material for: Magnetothermal nanoparticle technology alleviates parkinsonian-like symptoms in mice
Source: Nat Commun. 2021 Sep 22;12:5569. doi: 10.1038/s41467-021-25837-4 (PMC8458499; doi:10.1038/s41467-021-25837-4)
Supplement: Supplementary file 1 — Supplementary Information [file 41467_2021_25837_MOESM1_ESM.docx]

**Supplementary Information**

To consider the spatial extent of expected magnetothermal stimulation around the ferrofluid droplet, we employed a computational model. This model has two separate components: A) numerically solved heat transfer equations to predict the temperature expected in the vicinity of the droplet and B) application of a thermodynamic model of TRPV1 to predict the fraction of open TRPV1 channels that result at a given temperature.

To predict the time-dependent temperature field around the droplet, we follow the approach previously described for predicting magnetothermal stimulation via bulk heating ^1^. Crucially, the heat sinking term of Penne’s Bioheat equation is included to account for the effect of active perfusion of the tissue with blood at temperature $T_{b}$. In brief, we consider heat transfer equations in two regions: the droplet (region 1) and the surrounding tissue (region 2). Assumed quantities for relevant properties are provided in Table S1.

**Table S1:** Values of physical quantities used in heat transfer model.

| **Symbol** | **Description** | **Assumed Value** | **Units** | **Source** |
| --- | --- | --- | --- | --- |
| $\rho_{B}$ | brain tissue density | 1065 | kg m^-3^ | ^1^ |
| $c_{B}$ | brain specific heat capacity | 3630 | J kg^-1^ K^-1^ |  |
| $\rho_{b}$ | blood density | 1050 | kg m^-3^ |  |
| $c_{b}$ | blood specific heat capacity | 3617 | J kg^-1^ K^-1^ |  |
| $\omega_{b}$ | blood flow rate | 0.004 | s^-1^ |  |
| $K_{B}$ | brain thermal conductivity | 0.51 | W m^-1^ K^-1^ |  |
| $K_{b}$ | blood thermal conductivity | 0.52 | W m^-1^ K^-1^ |  |
| $T_{b}$ | arterial blood temperature | 310.15 | K |  |
| $h$ | effective heat transfer coefficient | 12 | W m^-2^ K^-1^ |  |
| $\rho_{d}$ | droplet density | $\rho_{d}c_{d}\approx\rho_{B}c_{B}$ | kg m^-3^ | approximation |
| $c_{d}$ | droplet specific heat capacity |  | J kg^-1^ K^-1^ |  |
| $K_{d}$ | nanoparticle droplet thermal conductivity | $K_{d}\approx K_{B}$ | W m^-1^ K^-1^ |  |
| $SLP$ | Specific loss power at relevant alternating magnetic field conditions | 558 | W/g_Fe_ | this work |
| $\gamma_{MNP}$ | concentration | 0.080  (8×10^4^) | g_Fe_ mL^-1^  (g_Fe_ m^-3^) |  |
| $r_{drop}$ | radius of droplet | 5×10^-4^ | m |  |
| $t_{on}$ | Duration of field application | 180 | s |  |
| $t_{lead}$ | lead in time before field application in simulation | 15 | s |  |
|  |  |  |  | arbitrary |

Heat transfer can be described in each region with a partial differential equation. In region 1 ($r<r_{drop},$ time dependent heat dissipation from nanoparticles is present, and there are no arterial heatsink effects),

|  | $\rho_{d}c_{d}\frac{\partial T_{1}}{\partial t}=K_{d}\nabla^{2}T_{1}+Q(t)$ | (S1) |
| --- | --- | --- |

In region II ($r>r_{drop}$, there is no heat dissipation from nanoparticles, and arterial heatsink effects are included),

|  | $\rho_{B}c_{B}\frac{\partial T_{2}}{\partial t}=K_{B}\nabla^{2}T_{2}+\rho_{b}c_{b}\omega_{b}\left( T_{b}-T_{2} \right)$ | (S2) |
| --- | --- | --- |

Because spherical symmetry is assumed, these equations reduce to one-dimensional radial forms. For region 1,

|  | $\rho_{d}c_{d}\frac{\partial T_{1}}{\partial t}=K_{d}\frac{1}{r^{2}}\frac{\partial}{\partial r}\left( r^{2}\frac{\partial T_{1}}{\partial r} \right)+Q(t)$ | (S3) |
| --- | --- | --- |

For region 2,

|  | $\rho_{B}c_{B}\frac{\partial T_{2}}{\partial t}=K_{B}\frac{1}{r^{2}}\frac{\partial}{\partial r}\left( r^{2}\frac{\partial T_{2}}{\partial r} \right)+\rho_{b}c_{b}\omega_{b}\left( T_{b}-T_{2} \right)$ | (S4) |
| --- | --- | --- |

To solve these equations numerically, we must establish relevant boundary conditions. For region 1, by geometric symmetry of spherical coordinates,

|  | $\left. \frac{\partial T_{1}}{\partial r} \right\vert_{r\to0}=0$ | (S5) |
| --- | --- | --- |

Enforcing heatflow at the outer edge of region 2,

|  | $\nabla T_{2}\left( t,r_{boundary} \right)=\frac{-h\left[ T(t,r_{boundary})-T_{b} \right]}{K_{B}}$ | (S6) |
| --- | --- | --- |

Between the two regions, heatflow must also be enforced.

|  | $K_{d}\nabla T_{1}(t,r_{drop})=K_{B}\nabla T_{2}(t,r_{drop})$ | (S7) |
| --- | --- | --- |

It is reasonable to approximate $K_{d}\approx K_{B}$, since the magnetic nanoparticles make up a small volume fraction of the droplet, and the droplet spends ample time in the brain, allowing for diffusion of biomolecules from the surrounding physiological milieu. (Differences in thermal conductivity between the regions are expected to be negligible.) This simplifies the boundary conditions between the regions as follows:

|  | $\left. \frac{\partial T_{1}}{\partial r} \right\vert_{r=r_{drop}}=\left. \frac{\partial T_{2}}{\partial r} \right\vert_{r=r_{drop}}$ | (S8) |
| --- | --- | --- |

This is simply a statement of continuity of the first derivative of the temperature function. If the approximation that $\rho_{d}c_{d}\approx\rho_{B}c_{B}$ is maintained from the previously reported model, it is possible to recast equations S3 and S4 as a single equation for both regions.

|  | $\rho_{d}c_{d}\frac{\partial T}{\partial t}=K_{d}\frac{1}{r^{2}}\frac{\partial}{\partial r}\left( r^{2}\frac{\partial T}{\partial r} \right)+Q\left( t \right)\left[ 1-H\left( r-r_{drop} \right) \right]$  $+\rho_{b}c_{b}\omega_{b}\left( T_{b}-T \right) H\left( r-r_{drop} \right)$ | (S9) |
| --- | --- | --- |

Here, $H$ is the Heaviside step function. The time dependence of $Q\left( t \right)$ can similarly be expressed in terms of Heaviside functions.

|  | $Q\left( t \right)=\gamma_{MNP} SLP \left[ H\left( t-t_{lead} \right)-H\left( t-t_{lead}-t_{on} \right) \right]$ | (S10) |
| --- | --- | --- |

The boundary conditions noted previously can be enforced at $r=0$ and the outer edge of the solution space. An initial condition of uniform temperature is assumed:

|  | $T\left( 0,r \right)=T_{b}$ | (S11) |
| --- | --- | --- |

The numerical solution to this heat transfer equation, solved with MATLAB is shown in Figure S1.


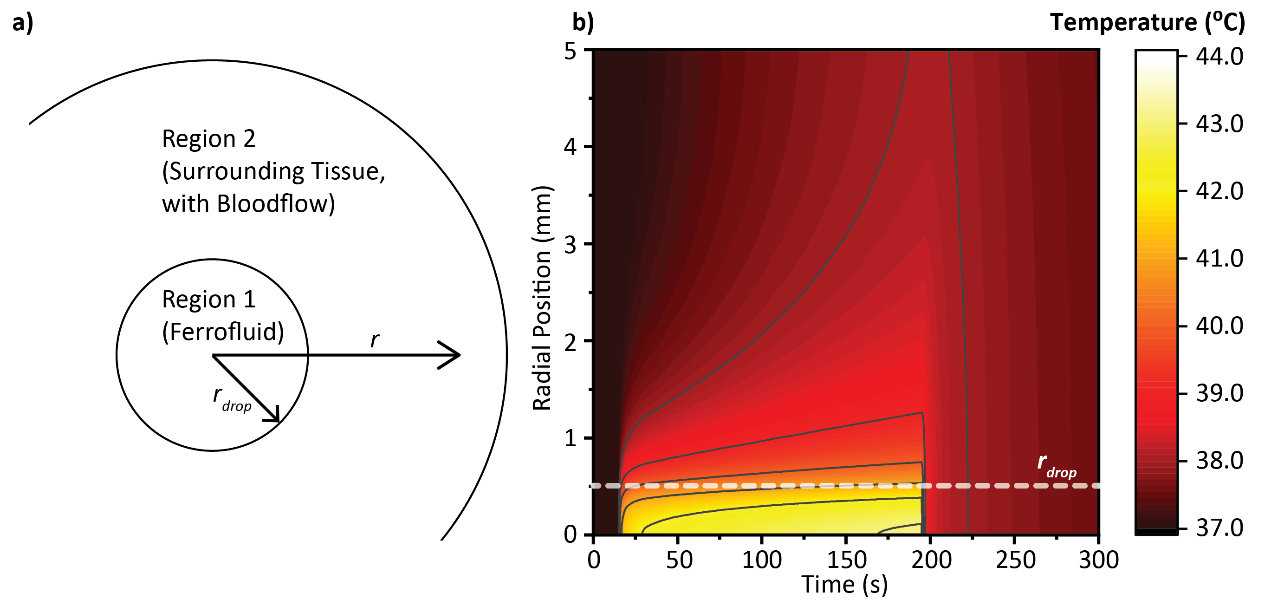


**Fig. S1:** Prediction of the temperature increase resulting around the injected nanoparticles subject to stimulation conditions used in this study. a) A schematic representation of the solution space considered by the partial differential equations explained in the text. Region 1 is within the spherical droplet of injected ferrofluid, whereas region 2 comprises the surrounding tissue. b) MATLAB was used to numerically solve for temperature as a function of time and radial position. The dashed line indicates the division between regions 1 and 2.

A previous experimental study of the threshold of activation for neurons overexpressing TRPV1 found a midpoint of activation at about 38 °C, with full activation at 39 °C ^2^. The extent of activation depends not only on the intrinsic properties of TRPV1 but also on extrinsic circumstances including the extent of expression of and endogenous positive feedback within the neurons. Nevertheless, the temperature threshold in that work was determined for a substantially similar system and can be applied here realistically. Assuming an activation threshold of 39°C for TRPV1 Fig S1b suggests that neurons within about 1 mm of the center of the droplet, or about half that distance from the surface of the droplet, are expected to reach this temperature during the course of stimulation. We note that neurons and brain tissue are not expected to reside within the radius of the droplet. Deviations from a spherical shape would increase the surface area to volume ratio of the droplet, increasing heat transfer efficiency and somewhat lowering temperature values inside and near the droplet. Moreover, densely concentrating MNPs can alter their effective SLP, either lowering or raising it ^3^.

To examine intrinsic effects on nearby TRPV1, it is possible to make use of a thermodynamic model that has been used to explain the temperature sensitivity of TRPV1 and to fit experimental characterization data ^4, 5^.

It uses a simple two state rate theory model to derive expressions for the equilibrium fraction of open channels. The rate of transition to from the closed state to the open state $\alpha$, is given by

|  | $\alpha=A\exp\frac{-E_{a,open}}{RT}\exp\frac{\delta zFV}{RT}$ | (S12) |
| --- | --- | --- |

Following that work, $E_{a,open}$ is the energy barrier to opening ($208 kJ/mol$), $R$ is the ideal gas constant ($8.314 J/mol/K$), $T$ is the absolute temperature ($310.15 K$), $\delta$ is the fraction of the gating charge moved in the outward direction ($0.5$), $z$ is the valence of the gating charge ($0.71$), $F$ is the Faraday constant ($9.649 \times{10}^{4} C/mol$), and $V$ is the transmembrane voltage. $A$ was a fitting parameter determined experimentally to be $1.61\times{10}^{37} 1/s$. Similarly, the rate of transitions from the open state to the closed state $\beta$ is given by

|  | $\beta=B\exp\frac{-E_{a,close}}{RT}\exp\frac{-(1-\delta)zFV}{RT}$ | (S13) |
| --- | --- | --- |

Here, the energy barrier to closing $E_{a,close}$ is $23.2 kJ/mol$) and the fitting parameter $B$ is $9.67\times{10}^{5} 1/s$. All other quantities remain the same. The equilibrium value for the fraction of open channels $P_{0}$, assuming timescales much longer than the attempt rates of individual channels, is given by

|  | $P_{0}=\frac{\alpha}{\alpha+\beta}.$ | (S14) |
| --- | --- | --- |

It is notable that $P_{0}$ depends on transmembrane voltage, although it is far less sensitive than voltage gated ion channels due to its smaller gating charge. Because $P_{0}$ increases with membrane depolarization as well as with temperature (Fig S2a), positive feedback is likely to contribute to this system, depending ultimately on the surface density of expressed TRPV1 channels among other factors. To consider the influence on neurons remaining at a resting potential of -70 mV, Fig S2b shows how the calculated temperature versus time field would influence $P_{0}$ at this potential. The fractional increase in $P_{0}$ from the -70 mV 37°C base state should be proportional to the increase in ionic current permitted by TRPV1 in response to elevated temperature. To consider the range of fractional increases that might be anticipated if positive feedback played a role, the case of -70 mV (resting potential) is shown as a lower bound, and the case of depolarization at 0 mV is taken as an upper bound (Fig S2c). The role of increased temperature even somewhat further from the droplet than the 39°C threshold may be to bias them toward activation.


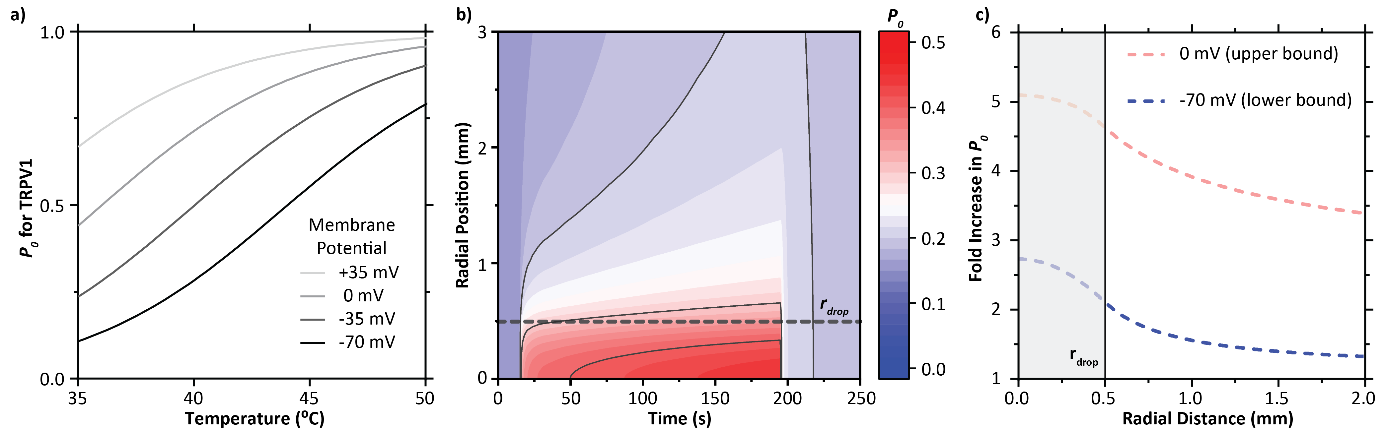


**Fig. S2:** The response of TRPV1 to temperature, in terms of the fraction of open channels *P_0_* is considered in the context of heat dissipated by the injected nanoparticles. a) Based on thermodynamic data from literature on TRPV1, expected *P_0_* versus temperature behavior is shown for several transmembrane potentials. b) Assuming a resting potential of about -70 mV, the time variation of *P_0_* is shown as predicted for the temperature distribution calculated in Fig S2b. c) The anticipated proportional increase in *P_0_* resulting from increased temperature as a function of distance from the injected droplet of magnetic nanoparticles is plotted. Because depolarization leads to increases in *P_0_*, positive feedback is likely and it is useful to consider the unperturbed -70 mV membrane potential as a lower bound and the depolarized case of 0 mV as an upper bound. Direct actuation is also limited to cells near enough to the transfection site to be transfected with TRPV1.

**Fig. S3:** Magnetic nanoparticles in brain tissue. Transmission electron micrographs of MNPs in the STN. Both images show nanoparticle solution in close proximity to neuronal cells. Scale bar is 200 nm.

**References**

1. Chen, R., Romero, G., Christiansen, M.G., Mohr, A. & Anikeeva, P. Wireless magnetothermal deep brain stimulation. *Science* **347**, 1477-1480 (2015).

2. Munshi, R. et al. Magnetothermal genetic deep brain stimulation of motor behaviors in awake, freely moving mice. *eLife* **6**, e27069 (2017).

3. Deatsch, A.E. & Evans, B.A. Heating efficiency in magnetic nanoparticle hyperthermia. *Journal of Magnetism and Magnetic Materials* **354**, 163-172 (2014).

4. Nilius, B. et al. Gating of TRP channels: a voltage connection? *The Journal of Physiology* **567**, 35-44 (2005).

5. Voets, T. et al. The principle of temperature-dependent gating in cold- and heat-sensitive TRP channels. *Nature* **430**, 748-754 (2004).
