# Supplementary material for: Magnetothermal nanoparticle technology alleviates parkinsonian-like symptoms in mice
Source: Nat Commun. 2021 Sep 22;12:5569. doi: 10.1038/s41467-021-25837-4 (PMC8458499; doi:10.1038/s41467-021-25837-4)
Supplement: Supplementary file 3 — Description of Additional Supplementary Files [file 41467_2021_25837_MOESM3_ESM.docx]

Description of Additional Supplementary Files

Title: Supplementary Movie 1

Description: Body-axis turning caused by mDBS in the STN. Representative video recordings of mouse injected with non-MNPs on the left and mouse injected with MNPs in the left STN on the right. The AMF is on for the duration of the video. Unilateral stimulation in the STN evokes contralateral rotations around the body axis. Video playback speed is 2X.
