# Supplementary material for: Magnetothermal nanoparticle technology alleviates parkinsonian-like symptoms in mice
Source: Nat Commun. 2021 Sep 22;12:5569. doi: 10.1038/s41467-021-25837-4 (PMC8458499; doi:10.1038/s41467-021-25837-4)
Supplement: Supplementary file 5 — Reporting Summary [file 41467_2021_25837_MOESM5_ESM.pdf]

nature research

Corresponding author(s): Sarah-Anna Hescham

Last updated by author(s): Aug 10, 2021

Reporting Summary

Nature Research wishes to improve the reproducibility of the work that we publish. This form provides structure for consistency and transparency in reporting. For further information on Nature Research policies, see our Editorial Policies and the Editorial Policy Checklist.

Statistics

For all statistical analyses, confirm that the following items are present in the figure legend, table legend, main text, or Methods section.

n/a	Confirmed	
☐	☒	The exact sample size (n) for each experimental group/condition, given as a discrete number and unit of measurement
☐	☒	A statement on whether measurements were taken from distinct samples or whether the same sample was measured repeatedly
☐	☒	The statistical test(s) used AND whether they are one- or two-sided<br>*Only common tests should be described solely by name; describe more complex techniques in the Methods section.*
☐	☒	A description of all covariates tested
☐	☒	A description of any assumptions or corrections, such as tests of normality and adjustment for multiple comparisons
☐	☒	A full description of the statistical parameters including central tendency (e.g. means) or other basic estimates (e.g. regression coefficient) AND variation (e.g. standard deviation) or associated estimates of uncertainty (e.g. confidence intervals)
☐	☒	For null hypothesis testing, the test statistic (e.g. F, t, r) with confidence intervals, effect sizes, degrees of freedom and P value noted<br>*Give P values as exact values whenever suitable.*
☒	☐	For Bayesian analysis, information on the choice of priors and Markov chain Monte Carlo settings
☒	☐	For hierarchical and complex designs, identification of the appropriate level for tests and full reporting of outcomes
☒	☐	Estimates of effect sizes (e.g. Cohen's d, Pearson's r), indicating how they were calculated

Our web collection on statistics for biologists contains articles on many of the points above.

Software and code

Policy information about availability of computer code

Data collection	Ethovision XT version 15, Noldus<br>StereoInvestigator version 10, Microbrightfield
Data analysis	SPSS version 25, IBM SPSS Statistics<br>Graph Pad Prism 8, Graph Pad Software<br>Custom MATLAB (version R2017a, MathWorks) scripts were used to analyze calcium activity for generation of ΔF/F0 signals. Heatmaps displaying ΔF/F0 vs. time were generated in Mathematica (version 11, Wolfram) software. All code is available upon request.

For manuscripts utilizing custom algorithms or software that are central to the research but not yet described in published literature, software must be made available to editors and reviewers. We strongly encourage code deposition in a community repository (e.g. GitHub). See the Nature Research guidelines for submitting code & software for further information.

Data

Policy information about availability of data

All manuscripts must include a data availability statement. This statement should provide the following information, where applicable:

- Accession codes, unique identifiers, or web links for publicly available datasets
- A list of figures that have associated raw data
- A description of any restrictions on data availability

Raw data associated with Figures 1-4 have been deposited in the GitHub database [https://github.com/shescham/magnetothermalDBS.git]

Field-specific reporting

Please select the one below that is the best fit for your research. If you are not sure, read the appropriate sections before making your selection.

- [x] Life sciences
- [ ] Behavioural & social sciences
- [ ] Ecological, evolutionary & environmental sciences

For a reference copy of the document with all sections, see nature.com/documents/nr-reporting-summary-flat.pdf

Life sciences study design

All studies must disclose on these points even when the disclosure is negative.

Sample size	Sample size was determined using a power calculation (calculated with G*Power, HHU Düsseldorf, Germany)
Data exclusions	No data was excluded from analysis
Replication	For the in-vitro study we have conducted a single experiment, out of which 30 cells were randomly selected. For the in-vivo study we evaluated the effect of mDBS between or within subjects across three different in-vivo experiments. All evaluations were successful.
Randomization	Cells were randomly selected for analysis and mice were randomly assigned to the different experimental groups.
Blinding	Investigators were blinded during analysis.

Reporting for specific materials, systems and methods

We require information from authors about some types of materials, experimental systems and methods used in many studies. Here, indicate whether each material, system or method listed is relevant to your study. If you are not sure if a list item applies to your research, read the appropriate section before selecting a response.

Materials & experimental systems

n/a	Involved in the study	
	x	Antibodies
	x	Eukaryotic cell lines
x		Palaeontology and archaeology
	x	Animals and other organisms
x		Human research participants
x		Clinical data
x		Dual use research of concern

Methods

n/a	Involved in the study	
x		ChIP-seq
x		Flow cytometry
x		MRI-based neuroimaging

Antibodies

Antibodies used	rabbit anti-c-Fos (K25), Santa Cruz Biotechnology Inc, RRID: AB_2231996, Catalog # sc-253<br>rabbit anti-tyrosine hydroxylase H-196, Santa Cruz Biotechnology Inc, RRID: AB_671397, Catalog # sc-14007<br>biotinylated donkey anti-rabbit, Jackson Immunoresearch Laboratories Inc, RRID: AB_2340593, Catalog # 711-065-152<br>donkey anti-rabbit Alexa Fluor 488, Invitrogen, RRID: AB_2535792, Catalog # A32790
Validation	The antibodies were validated in literature and in our laboratory for immunohistological staining of mouse brain.<br>rabbit anti-c-Fos (K25), RRID: AB_2231996, validated in PMID: 26597361<br>rabbit anti-tyrosine hydroxylase H-196, RRID: AB_671397, validated in PMID: 28602690

Eukaryotic cell lines

Policy information about cell lines

Cell line source(s)	HEK293FT (Thermo Fisher), gift from F. Zhang (MIT)
Authentication	Microscopic inspection
Mycoplasma contamination	This cell line is not mycoplasma contaminated
Commonly misidentified lines (See ICLAC register)	None

Animals and other organisms

Policy information about studies involving animals; ARRIVE guidelines recommended for reporting animal research

Laboratory animals	C57BL/6 male mice (Jackson Laboratory), 8 -10 weeks old at the time of first surgery
Wild animals	The study did not involve wild animals
Field-collected samples	The study did not include samples collected from the field
Ethics oversight	In-vivo experiment 1 was approved and carried out in accordance with Massachusetts Institute of Technology committee on Animal Care (protocol # 0713-063-16).<br>In-vivo experiment 2 and 3 was carried out under a protocol approved by the Institutional Animal Care Committee of Maastricht University in accordance to the Central Authority for Scientific Procedures on Animals (CCD; protocol # AVD1070020186046)

Note that full information on the approval of the study protocol must also be provided in the manuscript.
